# Hospital Meetings

**Published:** 1905-05-27

**Authors:** 


### HOSPITAL MEETINGS.

### CHELSEA HOSPITAL FOR WOMEN.

The 34th annual meeting of the Chelsea Hospital for Women was held at the institution on the 18th inst., Lord Glenesk being in the chair. The report was taken as read, a copy having been placed on every chair. A resolution was passed adopting the report and statement of accounts, also one to re-elect the council for the year.

Lord Glenesk, whom every one welcomed with enthusiasm on his reappearance amongst them after a severe illness, made a speech, drawing attention to the welcome fact that the hospital is in the rare position of being perfectly solvent, and indeed has a small sum in hand for what is called the "Emergency Fund." A large portion of this had been contributed by their generous friend, "H. M. E.," to whom they owed so much. Among other kind benefits which they received from her was that of the outside iron staircase for use in case of fire.

The annual subscriptions were well maintained; there was, however, no endowment, and it was greatly desired that generous friends and supporters would ere long manage to secure one, so as to relieve the management from anxiety as to the future. His lordship glanced at the admirable work done by the hospital, and the excellent rule that all patients really capable of paying some small sum towards their cure and maintenance should do so, whilst the truly indigent were received without any payment at all.

Lord Glenesk moved a vote of thanks to the medical and general staff, and also to the ladies' committee.

This was unanimously passed, and the meeting closed by Mr. J. S. Wood announcing that their kind and generous chairman had consented also to become their president. He moved a vote of thanks to his lordship for coming amongst them so soon after his illness and great sorrow at the loss of his brilliant and promising son.

### EAST END MOTHERS' HOME.

The annual meeting of the East End Mothers' Home was held at 15 Belgrave Square, by kind permission of the Duchess of Bedford, Dr. Owen Lankester, the chairman, presiding. The report was taken as read, and its adoption and the re-election of the honorary officers and the committee of management was moved by Mrs. Scharlieb, M.D., and seconded by Mr. Goodrich, L.C.C. The resolution was unanimously adopted.

Mrs. Scharlieb made an interesting speech. She had lately visited the Mothers' Home, and gave unqualified testimony to its excellent management, and also to the great necessity of extending its usefulness if possible by further financial help. A second resolution was passed, proposed by the honorary treasurer and seconded by the Hon. G. J. Goschen, M.P., that by all possible means the charity should be helped and extended. It is greatly hoped that by the generous help of its supporters the freehold of the next house may be bought, and the heavy rental thus saved. Amongst the smaller needs of the Home are a lift and furniture. Visitors would be gladly welcomed; and it is hoped that personal inspection will stimulate the desire to help in so excellent a charity.

Proceedings terminated with a vote of thanks to the Duchess of Bedford for lending her house to the meeting.

### NATIONAL SANATORIUM FOR CONSUMPTION, BOURNEMOUTH.

The Earl of Eldon presided at the 50th annual meeting of the governors of the National Sanatorium for Consumption and Diseases of the Chest, held at the Westminster Palace Hotel on the 11th inst. Among those present were the Right Hon. A. F. Jeffreys, M.P., General Stansfeld, Colonel A. D. Eden, Mr. G. J. Fenwick, Mr. Simeon Lazarus, and Mr. G. Barbour.

The annual report of the Committee of Management, which was unanimously adopted, stated that the past year had been one of great importance in the history of the institution, an extension of the building and considerable alterations and improvements having been carried out by which 14 more beds had been added for patients, and more adequate and suitable accommodation provided for the nursing staff. The cost of these works, including furnishing, had amounted to over £3,000, towards which only £2,000 had been subscribed. The ordinary income for 1904 amounted to £2,999, and the ordinary expenditure to £3,094. The latter, however, was abnormally low owing to the unavoidable closing of the sanatorium for four months in order to carry out the above extension and improvements, so that a large increase in the income is essential to enable the committee to continue to keep the whole of the institution open. The report further stated that with the increasing pressure for admission from all parts of the kingdom, the committee were most reluctant to close any portion of the sanatorium, and they were therefore making a special appeal for new annual subscriptions to meet the current expenditure, and for donations to pay off the total debt of £1,500. They were anxious to commemorate the Jubilee year of the institution by placing it in a sound financial condition.

The account of the year's work showed that the results of the open-air treatment continued to be very satisfactory. Nearly 300 patients were under treatment, a very large proportion of whom were discharged considerably improved.

The Duke of Portland, K.G., the Marquess of Salisbury, the Marquess of Zetland, K.T., Lord Eustace Cecil, the Bishop of Salisbury, Lord Rothschild, the Hon. T. A. Brassey, and the Right Hon. Arthur F. Jeffreys, M.P., were elected vice-presidents.

Mr. H. E. Acklom, Colonel A. D. Eden, Mr. D. H. W. Robson-Burrows, General H. H. Stansfeld, and Mr. F. Stokes were re-elected members of the Committee of Management. The following were also elected to serve on the Committee:—Major K. R. Balfour, M.P., Mr. Walter C. Clark, Mr. W. Gibson, and Mr. Simeon Lazarus.

Upon the recommendation of the Committee it was resolved that in future donors of £500 shall have the right to name a ward and donors of £100 to name a bed, in perpetuity.

The meeting terminated with a cordial vote of thanks to Lord Eldon for his kindness in presiding.

